# Monitoring consumption Switzerland: data, background, and use cases

**DOI:** 10.1186/s41937-023-00108-9

**Published:** 2023-03-20

**Authors:** Martin Brown, Matthias R. Fengler, Jonas Huwyler, Winfried Koeniger, Rafael Lalive, Robert Rohrkemper

**Affiliations:** 1 University of Lausanne, Lausanne, Switzerland; 2 University of St. Gallen, St. Gallen, Switzerland; 3 Study Center Gerzensee, Gerzensee, Switzerland; 4 Wordline, Zurich, Switzerland

**Keywords:** Monitoring, Consumption, Payment data, COVID-19, D12, E21, E65

## Abstract

Monitoring Consumption Switzerland is a public–private partnership between the University of St. Gallen and the payment companies Worldline and SIX that processes and publishes payment data on transactions in Switzerland processed by Wordline/SIX in real time. This paper provides background information on this novel source of data and presents their attributes, aggregation and granularity, and their interpretability. The paper presents several use cases that show the strengths of the data, and it alerts future users of the data to possible challenges. The paper also discusses the project's impact and provides an outlook.

## Introduction

Economic analysis and policy decisions require accurate information that is updated frequently and ideally available in real time. This is particularly valuable in times of crisis when large changes of economic fundamentals occur at high frequency. In the modern economy, private-sector firms own a wealth of granular high-frequency data. These data have the potential to improve economic policy decisions and inform the general public once they are made publicly available in an anonymized form.

This basic truth became particularly conspicuous when the COVID-19 pandemic hit the world and policy makers around the globe scrambled for the right measures to fight the imminent health crisis without choking economic activity. In the light of this situation, the authors launched the initiative Monitoring Consumption Switzerland (MCS) as a private–public partnership between the University of St. Gallen (HSG) and the two major Swiss payment service providers SIX and Worldline, both headquartered in Zürich. MCS receives transaction data on electronic payments and cash withdrawals of Swiss debit card holders in Switzerland and abroad as well as electronic payments by Swiss and foreign card holders at merchants in Switzerland made by means of debit cards, credit cards, and mobile payment wallets. The data thus comprise both an issuing data perspective, i.e., the debit card holder perspective, and an acquiring data perspective, i.e., the merchant/payment terminal perspective of the consumer transaction data. MCS shares the data with the public on the website https://monitoringconsumption.com/ with a delay of one week.^1^

This paper provides the background to and detailed information on these data relevant to current and potential future users of its data. As SIX and Worldline cover a significant market share of the Swiss payment service markets, the data cover a considerable amount of private consumption in Switzerland, which we estimate to amount to about one quarter of the Swiss GDP. The data are available at a daily frequency and published weekly. To ensure anonymity of card holders and merchants, the transaction data are aggregated and scaled. They are sliced along geographical regions (the 26 Swiss cantons or 7 larger regions), along 7 levels of urban agglomeration and along 12 merchant categories (based on NOGA codes). Data coverage starts in January 2019, i.e., a year before the pandemic, and the current agreements ensure data updates at least throughout 2022. Due to this sectoral and geographical segregation, their high-frequency, and regular updates, the data have delivered immediate insights, for example, into how consumer spending has responded to the various policy measures enacted to curb the spread of COVID-19.

MCS is among many initiatives and projects that have been launched in the wake of the COVID-19 pandemic to monitor and track economic activity. As regards Switzerland, Kraenzlin et al. (2020) have been among the first to analyze the impact of the COVID-19 crisis on payment behavior in the Swiss retail sector. Carvalho et al. (2021) analyze credit and debit card data mediated through one of the largest banks in Spain; similarly, Andersen et al. (2020) study payments with card and mobile wallets, cash withdrawals and incoming money flows obtained from a large Danish retail bank. A more holistic view on electronic payment transactions is offered by Byrne et al. (2020) as regards the Irish economy by accessing the Credit and Debit Card Statistics of the Central Bank of Ireland, while Ardizzi et al. (2020) work with data obtained from the retail settlement system managed by the Bank of Italy; a similarly comprehensive data source is employed in Bounie et al. (2020) who study the card payment scheme of the French “carte bancaire” system. Besides traditional banks, Fintech start-ups also provide payment transaction data. For instance, Hacioglu et al. (2020) look at data of debit and credit card transaction data linked to U.K.’s largest personal financial manager.

As for North America, Dahlhaus and Welte (2021) analyze debit and credit payment data gathered from two Canadian payment service providers. As regards the USA, Baker et al. (2020) use data obtained through a US personal financial manager to study user spending and income transactions from all linked checking, savings, and credit card accounts. The possibly most comprehensive data on US real-time consumption data are procured by Chetty et al. (2020) who collect credit and debit card spending and cash spending of US consumers. These data are further complemented by data on small business transactions and revenues, payroll data, job postings, educational data as well as mobility data. Besides this, a number of central banks and economic research institutes developed broader real-time measures of economic activity; examples include the weekly GDP trackers of the Deutsche Bundesbank for Germany,^2^ of the OeNB for Austria^3^ and the SECO for Switzerland.^4^ All these studies and indicators document that significant drops in consumption and economic activity are associated with the tightness of the regional lockdown measures and the extent of social distancing policies.

The paper is organized as follows. We discuss the data sources in Sect. 2 and the data sets we publish in Sect. 3. Section 4 provides information on data quality assurance. Section 5 suggests approaches to validate the project data and Sect. 6 provides use cases. Section 7 concludes.

## Data sources

Information on consumer spending and payment behavior can be elicited through survey methods or can be sourced through the secondary use of administrative data. Consumer budget surveys (reference) elicit all non-recurring and recurring transactions of a sample population for a limited period of time–usually one month. These surveys typically provide information on the product or service purchased, as well as the expenditure amount. Payment diary surveys elicit non-recurring spending over a shorter period of time–usually 3 to 7 days. These surveys commonly elicit the location of purchase and method of payment (cash, card, mobile, etc.), but no information on the specific product or service purchased. In both cases, data are available only for a small sample of consumers over a very limited period of time. The secondary use of administrative data allows researchers to observe spending and payment behavior for a larger sample of consumers over a longer period of time. Sources of administrative data can be banks (Aastveit et al., 2020; Brown et al., 2021), card issuing companies (Gathergood & Guttman-Kenney, 2021), merchants (Klee, 2008), financial aggregators (Olafsson & Pagel, 2018) or the service providers, which process payment transactions.

### Payment transaction data: acquiring and issuing data

The payment data published by MCS provide two complementary views of consumption activity in Switzerland: on the one hand, there is the merchant view; on the other hand, the cardholder view. Importantly, the two data sources cover different scopes of transactions, and therefore enable different types of analyses.

The merchant view is engrained in the *acquiring data*, which provide a comprehensive record of all (electronic) transactions made at a payment terminal, irrespective of the type and owner of the payment instrument. These data thus cover transactions made by debit card, credit card, or mobile payment apps^5^ conducted by both domestic and foreign consumers. The cardholder view instead is given by the *issuing data*. These data cover all transactions conducted on all debit cards issued by a large sample of domestic banks. Considering electronic payments at payment terminals in Switzerland, these data thus include transactions of domestic consumers on one type of payment instrument only. In addition, the issuing data provide information on ATM transactions conducted with the same debit cards in Switzerland. Moreover, the issuing data cover electronic payments and ATM transactions made with the same cards outside of Switzerland.

The two different data types published by MCS are provided by two payment service providers. Worldline procures the acquiring data to the project. Their data cover all electronic payments made at point of sale (PoS) or ecommerce terminals of all merchants, which are domiciled in Switzerland and in the merchant acquiring portfolio of Worldline. A comparison with aggregate data published by the Swiss National Bank suggests that the acquiring data provided to the project cover the large majority of electronic transactions made at domestic merchants.^6^

SIX contributes the debit card issuing data to the project.^7^ These data cover all PoS payments and ATM transactions by debit card for all clients of the banks, which have their debit card transactions processed by SIX. A comparison with aggregate data published by the Swiss National Bank confirms that the issuing data provided to the project covers a clear majority of all debit card transactions in Switzerland. Payment survey data confirm that cash and debit cards account for 57% of all non-recurring spending of Swiss consumers in 2020, whereby cash is almost exclusively obtained through debit card ATM withdrawals (Swiss National Bank, 2021a, 2021b).

**Fig. 1:**
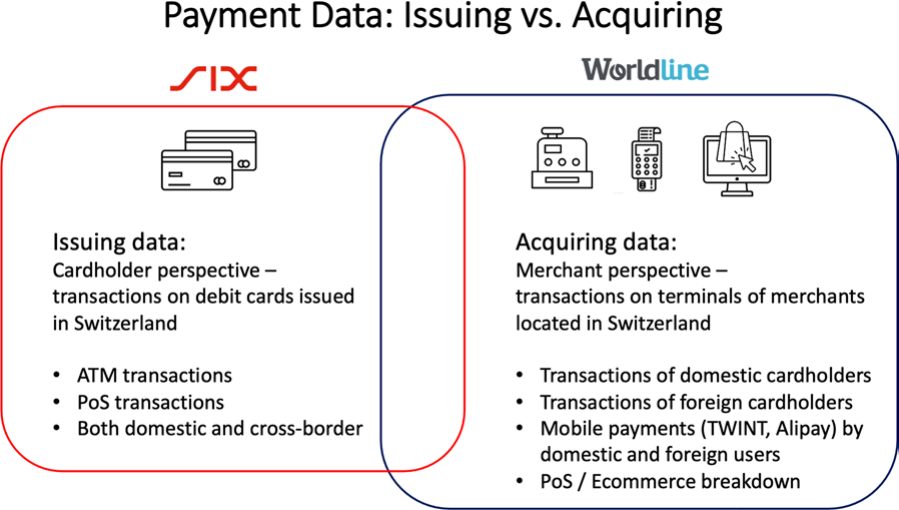
Payment processing data: acquiring vs. issuing. *Note*: The figure displays the origin of the transaction data. The data originate from two sources: Transactions recorded when generated by specific debit cards, provided by SIX (card issuer perspective); and transactions from arbitrary payment cards recorded at the payment terminal, provided by Worldline (acquiring perspective)

Figure 1 illustrates the two complementary views of consumption activity in Switzerland as provided by the two data sources. The overlapping area in Fig. 1 indicates that both data sets contain debit card transactions. Because we provide both data sets separately, this overlap in coverage does not create an issue of double-counting. Data users should not merge the data from the different sources because the extent of the overlap is not known and the sum of transactions in both data sets, for example, would be meaningless.

### Raw data attributes

For both the acquiring data and the issuing data, MCS publishes daily aggregated data. Data at the transaction level are not provided due to reasons of data privacy. We provide details on the level of aggregation in the section on data aggregation below.

The raw data underlying the published daily data are transaction-level information, processed by SIX and Worldline for their clients, i.e., debit card issuing banks and merchants respectively. For each payment transaction, our data partners process information on the date/time of the transaction, the value of the transaction (in CHF) as well as information on the payment terminal (or ATM) and payment instrument involved.

Regarding the *payment terminal*, the raw acquiring data provide information on the payment channel (PoS or ecommerce), the geographical location of the merchant (address, postcode), and the category of the merchant. Payment service providers worldwide adhere to a uniform classification of merchants (MCC = merchant category code), which can be mapped to standard industry classifications (NACE, or in the Swiss case NOGA). The issuing data additionally identify transactions, which are made at ATM as opposed to electronic payment channels.

Regarding the *payment instrument* involved in a transaction, the raw transaction data include information on the payment instrument type (debit card, credit card, mobile app) as well as the country in which the instrument was issued. For the issuing data, the payment instrument is always a debit card and the issuing country is always Switzerland (Table 1).

**Table 1:** Overview of MCS acquiring and issuing data

	Acquiring data	Issuing data
Merchants	Selected merchants population	All merchants—> domestic and foreign
Cardholders	All cardholders—> domestic and foreign	Selected customer population
Payment instruments	All payment instruments	1 payment instrument
Cash transactions	n/a	ATM transactions
Merchant info	Category, location, channel	Category, location, channel
Consumer info	Limited	Limited
Product info	n/a	n/a

The table displays the different attributes available in the acquiring and issuing data. See Tables 2, 3 and 4 for more details on the data we provide

### Data aggregation

The University of St.Gallen maintains a data publishing agreement with Worldline and SIX, according to which the MCS project can publish daily aggregated acquiring data and debit card issuing data. The aggregation of the data ensures that the transactions of no individual merchant (or cardholder) is identifiable. In order to preserve the anonymity of individual merchants, the raw transaction-level data are aggregated by region and merchant category in particular. The corresponding aggregation is conducted by the data providers before it is transmitted to the University of St.Gallen team, which manages the project.

The *aggregation by merchant category* maps individual merchant category codes (e.g., 4751 = clothes retailer) into standard industry classification codes (e.g., NOGA4 = 4751) and then aggregates these industries according to the NOGA hierarchy. The MCS project publishes data aggregated for twelve separate merchant categories:^8^ Retail: Food, Beverage & Tobacco; Retail: Other goods; Retail Fuel Stations; Food & Beverage Services; Accommodation; Transport Services; Motor vehicles; Professional Services; Financial Services; Entertainment & Sports; Human Health Services; Personal Services. Merchants which cannot be classified in one of the above categories are bunched into a category “Other”. In the case that a particular aggregation does not preserve the anonymity of the underlying merchants, the observations in question are also grouped into the category “Other”.

The *aggregation by location* is conducted along two dimensions. A first (political) classification is based on NUTS statistical regions,^9^ where we distinguish locations at the NUTS-1 (country), NUTS-2 (*Grossregion*); and NUTS-3 (Canton) level. A second classification is into municipalities by agglomeration types based on the Swiss Federal Statistical Office (FSO) 2012 definition of areas with urban character.^10^ The FSO defines municipalities as cores of agglomerations based on the IJO (inhabitants, jobs, overnight stays) concept and allocates further municipalities to an agglomeration based on commuter flows to the core. We distinguish seven municipalities: ​​rural municipalities without urban character, agglomeration core municipalities: core cities, core municipality of agglomeration: primary core, core municipality of agglomeration: secondary core, municipalities oriented to multiple cores, core municipalities outside agglomeration. A further category *Other* contains all municipalities that cannot be assigned to the aforementioned categories.^11^

### Data scaling

In addition to the anonymization of the data by means of aggregation, the published transaction volumes in CHF and number of transactions are scaled by the MCS team before publication, according to the agreement with the project partners. To scale the data, we use the average daily turnover (in CHF) or the average daily number of transactions with all means of payment methods (incl. ATM withdrawals) in January 2020. We thus divide the actual value of sales by the average daily value of sales realized in January 2020 to obtain the *Scaled Value*. Analogously, we divide the actual number of transactions by the average daily number of transactions in January 2020 to obtain the *Scaled Number of Transactions*. Importantly, we normalize all value series and all number-of-transactions series by the *same* respective constant. This normalization ensures that the data are still informative about the relative turnover across different regions, merchant categories, and agglomeration types. This choice of scaling thus serves the purpose of anonymization while maintaining the interpretation of the data across expenditure categories or regions.

## Data sets

MCS publishes data sets based on daily acquiring and issuing data. An overview of the available data and links to the corresponding data files (in.csv) and data description files are provided on the project webpage. All published data sets are updated on a weekly basis only. The published data sets are hosted on the publicly accessible file system SWITCHdrive.

The project currently publishes nine data sets with different aggregations of the *acquiring data*. These data sets differ in the granularity of information about the payment terminal (merchant location, merchant category, channel) and payment instrument (type, origin). Table 2 in the appendix provides an overview of the available data sets. Each data set contains two daily measures for each combination of attributes: the *Scaled Value* and the *Scaled Number of Transactions*.^12^

The project currently publishes five data sets with different aggregations of the *issuing data*. These data sets differ in the granularity of information about the payment terminal (merchant location, merchant category, channel). By definition, the payment instrument (debit cards of domestic origin only) is constant for all of these tables. Table 3 in the appendix provides an overview of the available data sets. As for the acquiring data, each data set of the issuing data also contains two daily measures for each combination of attributes called *Scaled Value* and *Scaled Number of Transactions*.

The project publishes one overview data set (MCS_Overview_Data). These data provide a daily aggregate of transactions by payment channel (PoS, ecommerce, ATM), payment instrument type (debit, credit, mobile) and payment instrument origin (domestic, foreign) based on the acquiring and issuing data.^13^

**Fig. 2:**
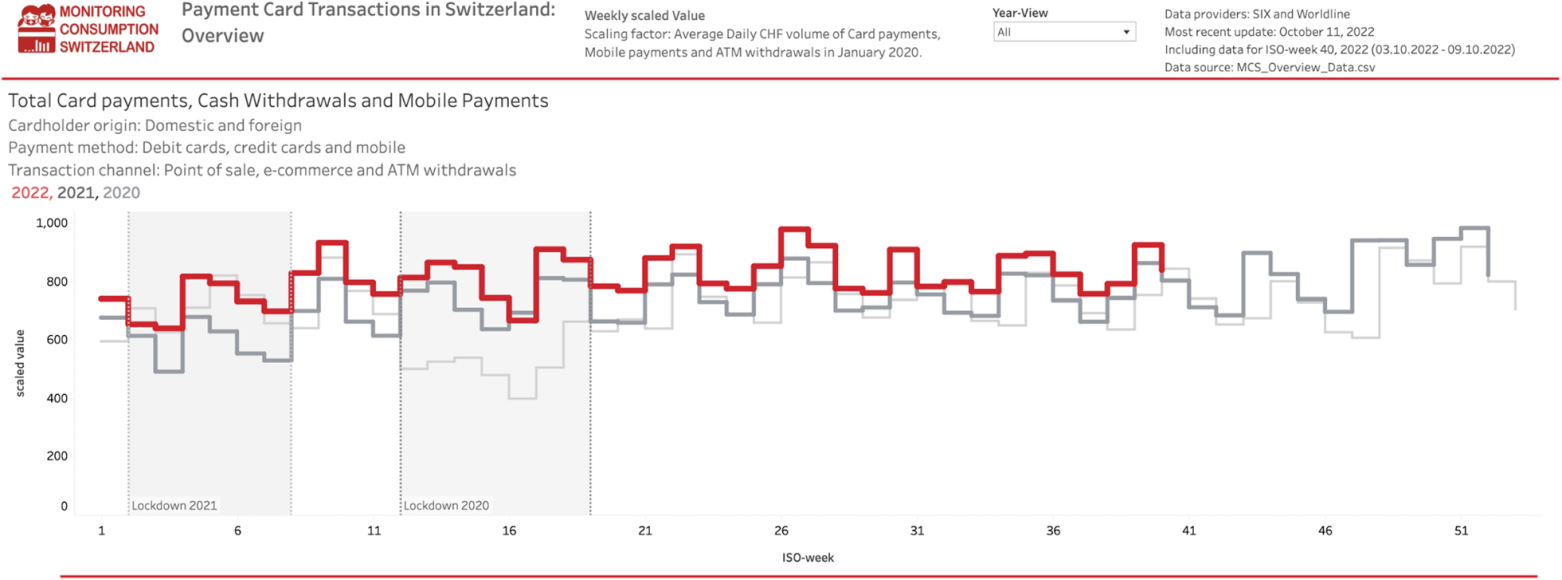
Example of data visualization. *Note*: The figure illustrates the development of consumer spending (debit & credit cards, mobile payments and ATM withdrawals) in 2020 (light gray), 2021 (dark gray) and 2022 (red). The data are aggregated on a weekly basis (see stair pattern). The first (spring 2020) and second (spring 2021) lockdowns are marked with shading. We display the data as provided, i.e., as a scaled value, which cannot be interpreted in its own right but which preserves the relative interpretability both over time and in the cross-section. See Sect. 2 for the details on the scaling. The most recent version of this (interactive) dashboard is available on the project website (see dashboard “Payment Card Transactions in Switzerland: Overview”). Data source: MCS_Overview_Data.csv

Data from the above-mentioned data sets are visualized on the project webpage using dashboards created with Tableau Public.^14^ The data visualizations are updated when new data are published and carry the date of the most recent data update. Figure 2 illustrates the overview dashboard as per October 09, 2022.

## Data quality assurance

MCS executes an automated data quality assurance procedure to ensure the consistency of the data. Inconsistencies might be caused in the process of data collection (e.g., software problems with terminals or payment cards), data processing, or data aggregation. Such issues would affect the economic interpretation of the data related to consumer spending or merchant turnover. The procedure we describe in the following only concerns the detection of non-economic, i.e., technical, irregularities that may have occurred during the data update. Typical examples include the delayed processing of transaction data or double countings. In such cases, the project team revises false data in close cooperation with the data providers. In the case of economic irregularities, however, the data remain as delivered. Economic irregularities may occur due to local events like fairs and cantonal holidays, or the introduction and revocation of the policy measures enacted to curb the pandemic. In line with the frequency of publication, the data quality assurance is conducted on a weekly basis before the data are made publicly available.

In detail, the procedure takes the form of an automated algorithm written in the programming language *R*, making use of the open-source packages *anomalize*^15^ and *forecast*.^16^ To enable a thorough analysis of the data, the algorithm initially decomposes each data set into multiple time series of its finest categorizations. For example, the ATM_Canton.csv data set is divided into a time series of daily ATM withdrawals and a time series of daily ATM deposits for each canton. Through this partitioning, each individual data series can be analyzed in detail without being obscured by an aggregation process.

For the algorithm to learn the structure of a time series, it considers a time span of three months.^17^ Within this time period, a trend and seasonal component (recurring weekday pattern) of the time series is found by a decomposition and used to reduce the time series to a residual component (detrended and deseasonalized time series). To further remove any linear dependence on the residual component that might have been missed by removing the trend and season, an autoregressive moving-average (ARMA) model is fitted. The residual series is subjected to an outlier detection test. More specifically, an extreme Studentized deviate test is used. This test sequentially performs outlier tests for a gradually increasing number of potential outliers at a confidence level of 95%, selects the most probable number of anomalies or irregularities and labels them accordingly. The test has the advantage that it determines the number of outliers one by one and thus sidesteps having to specify the number of outliers a priori.

If an irregularity is detected in the time series within the last seven days (period between subsequent outlier checks), it is recorded and visualized in an automated report. The report shows exactly in which subcategory of the data set an irregularity was detected.

Apart from detecting inconsistencies in the data, the procedure has a further benefit. While detecting erroneous inconsistencies, the standardized procedure also directly detects valid irregularities caused by true structural breaks in consumer spending. Such irregularities might be triggered by, for example, a public holiday, a pay day (ATM transactions) or the imposition of a lockdown. The quality assurance procedure thus not only verifies the validity of the data but also provides an initial indication of unusual events to the MCS team. The project currently runs the checks on the following four data sets ACQ_POS_Grossregion_NOGA.csv, ACQ_POS_Canton.csv, ATM_Canton.csv, DEBIT_Canton.csv.

**Fig. 3:**
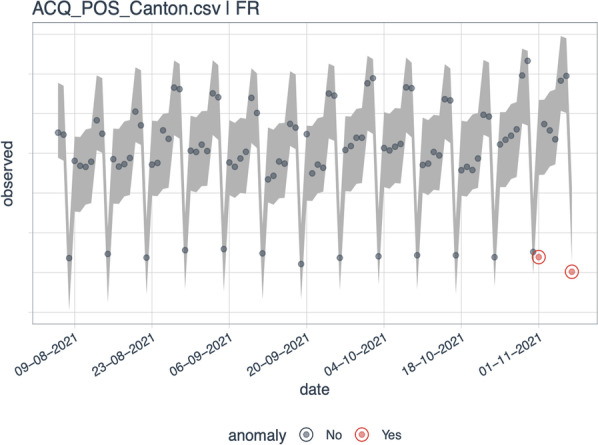
Example of irregularity detection. *Note*: The figure displays the time series of cashless payments at PoS in the canton of Fribourg (between 09-08-2021 and 07-11-2021). The gray border indicates the confidence limits, while the dots represent observations of a single day. Red dots indicate irregularities: the one on 01-11-2021 is an economic irregularity due to a local holiday; the one on the far right is due to the late processing of transactions occurring on the last day of the sample (a Sunday), which was corrected in a subsequent data update. Data source: ACQ_POS_Canton.csv

An example for a reported irregularity detection is shown in Fig. 3. The plot displays data coming from the file ACQ_POS_Canton.csv which contains accumulated cashless PoS payments (in CHF) categorized by Swiss cantons. In the specific time series shown in the figure, data points for the canton of Fribourg (FR) are plotted with two irregularities, indicated by two red points. The irregularities exceed the gray band indicating the daily 95% confidence intervals considering the trend and the day of the week pattern (assuming homoscedasticity). The first irregularity (red point from the left) was triggered by the fact that the 1st of November (known as All Saints' Day) is a public holiday in the canton of Fribourg. Since this public holiday was a Monday in 2021, this led to an irregularity because of a significant drop in PoS transactions due to closed stores. It is thus an economic irregularity which is not revised. The second irregularity was caused by the fact that the processing of the transaction data of the last day (Sunday) in the data set was delayed for some terminals in the canton of Fribourg. Therefore, not all transactions of this day were registered at the time of data collection. This technical data irregularity was revised in the subsequent data update.

## Data validation

### Representativeness of the data

MCS features payment data processed at specified payment terminals in Switzerland or with specified debit cards issued in Switzerland. This means that the data merely entail a subsample of all Swiss cashless payments and ATM transactions. This raises the question of how representative the data is. We thus provide a comparison of the characteristics of transactions in the MCS data with other available data sources.

**Fig. 4:**
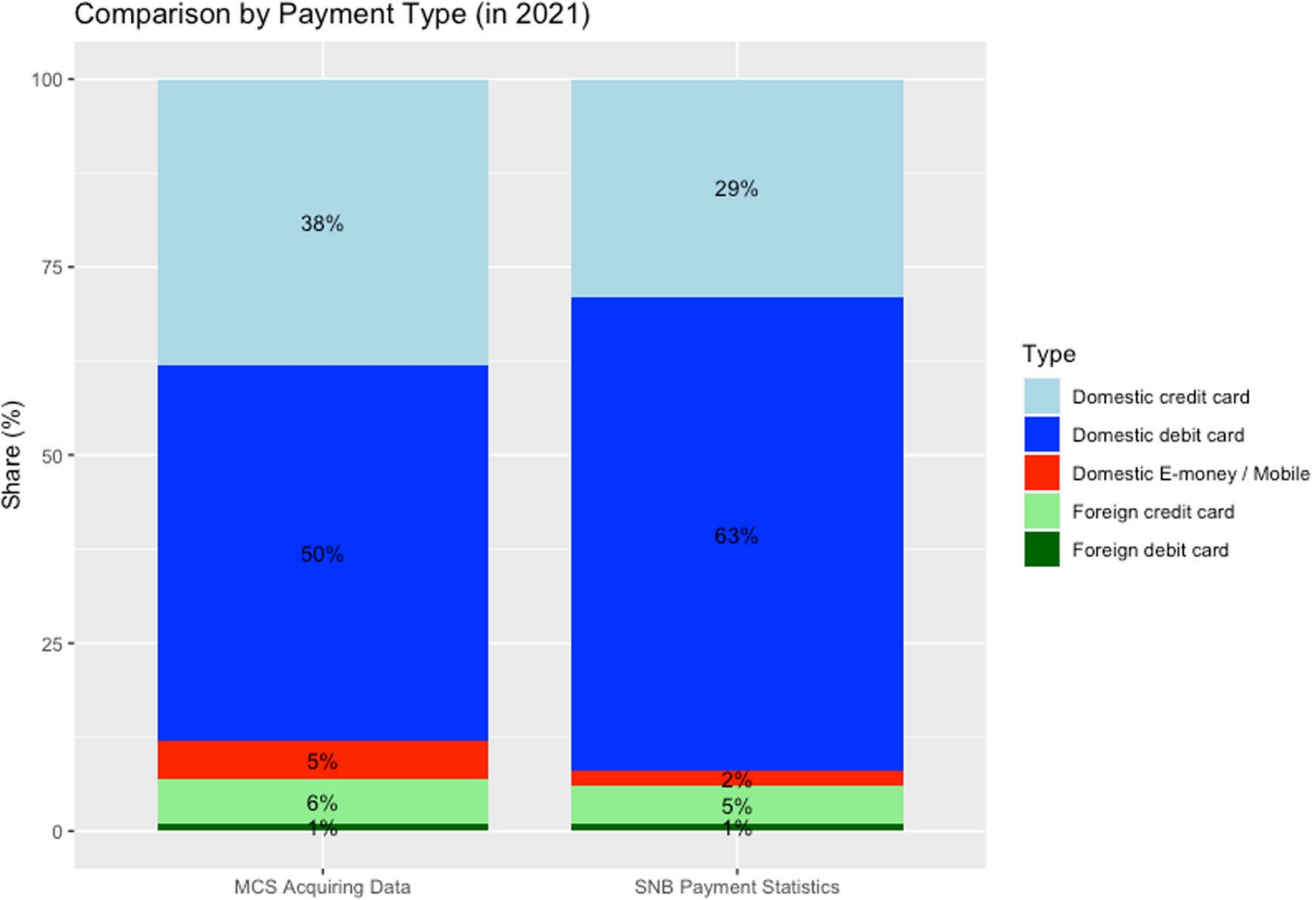
Comparison of MCS acquiring data to SNB payment statistics, 2021. *Note*: The figure displays the distribution of different payment methods in the acquiring data against the official distribution of payment methods in Switzerland according to the SNB. Data source: MCS_Overview_Data.csv, SNB *Payment and Cash Withdrawals* (https://data.snb.ch/en/topics/finma/cube/zavezaluba)

The Swiss National Bank (SNB) publishes aggregate, monthly statistics on cashless payment transactions from the perspective of the acquirer and the issuer, as well as statistics on ATM transactions by Swiss debit cards on a national level.^18^ At the monthly level, the project data thus can be compared with overall payment transaction data published by the SNB (Swiss National Bank, 2021a). In Fig. 4, we compare the MCS acquiring data to that reported by the Swiss National Bank for the year 2021. First, we compare the structure of the payments made in Switzerland during 2021 by cardholder origin (domestic vs. foreign) and payment instrument (credit card, debit card, mobile). The acquiring data reported by MCS compares well to the aggregated statistics reported by the SNB. Domestic debit cards and credit cards account for roughly 90% of the transaction value in both data sets. It is worth noting that domestic debit (credit) cards account for a lower (higher) share of the transaction value in the MCS acquiring data compared to the SNB data. This is likely due to that the MCS data do not cover transactions of Postfinance cards, which are counted as debit cards.

**Fig. 5:**
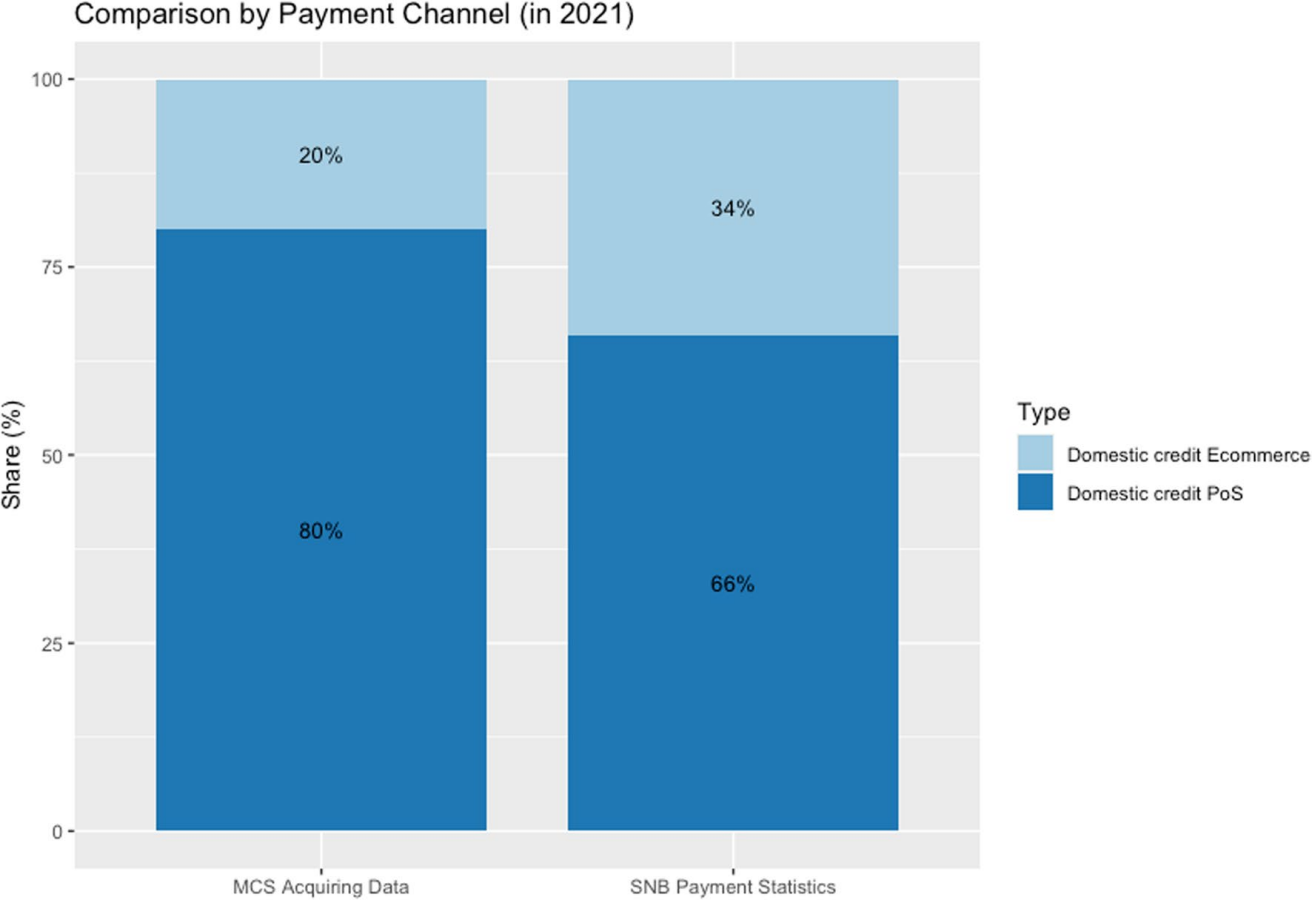
Comparison of MCS acquiring data to SNB payment statistics, 2021. *Note*: The figure displays the distribution of payment channels in the acquiring data against the official distribution of payment channels in Switzerland according to SNB. Data source: MCS_Overview_Data.csv, SNB *Payment and Cash Withdrawals* (https://data.snb.ch/en/topics/finma/cube/zavezaluba)

Considering domestic credit card transactions only, we compare the MCS acquiring data and the SNB statistics regarding the payment channels. Figure 5 shows a larger share of PoS transactions compared to ecommerce transactions in the MCS acquiring data than in the SNB aggregate statistics.^19^ Some ecommerce transactions (in particular related to travel) are less likely to be covered by our data provider.

**Fig. 6:**
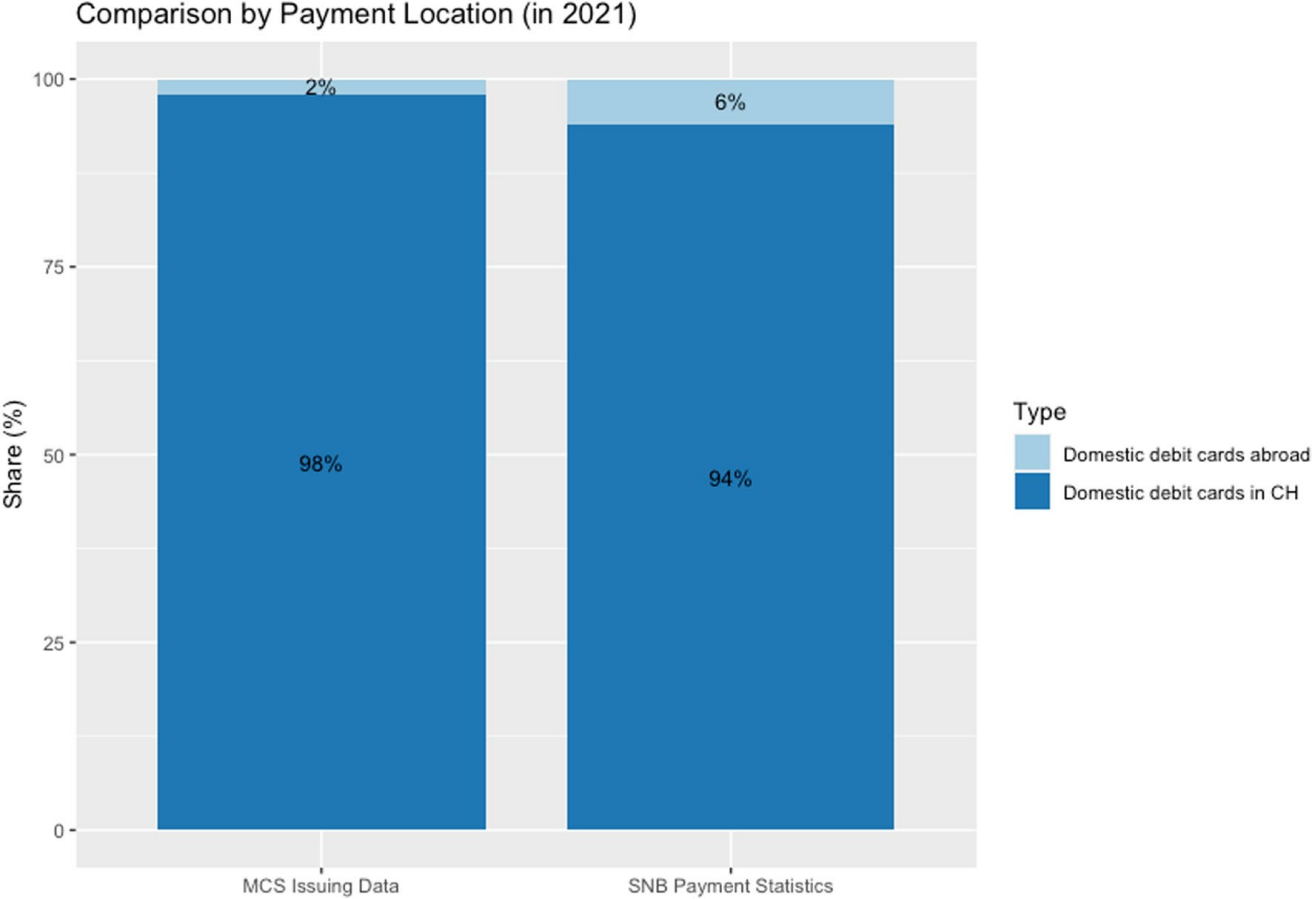
Comparison of MCS issuing data to SNB payment statistics, 2021. *Note*: The figure displays the location of debit card transactions in the issuing data against the location of debit card transactions according to SNB. Data source: MCS_Overview_Data.csv, SNB «Payment and Cash Withdrawals.» (https://data.snb.ch/en/topics/finma/cube/zavezaluba)

In Fig. 6, we compare the MCS issuing data regarding the location of the debit card transactions. Figure 6 shows a slightly larger share of domestic transactions in the MCS issuing data relative to the SNB aggregate statistics.

**Fig. 7:**
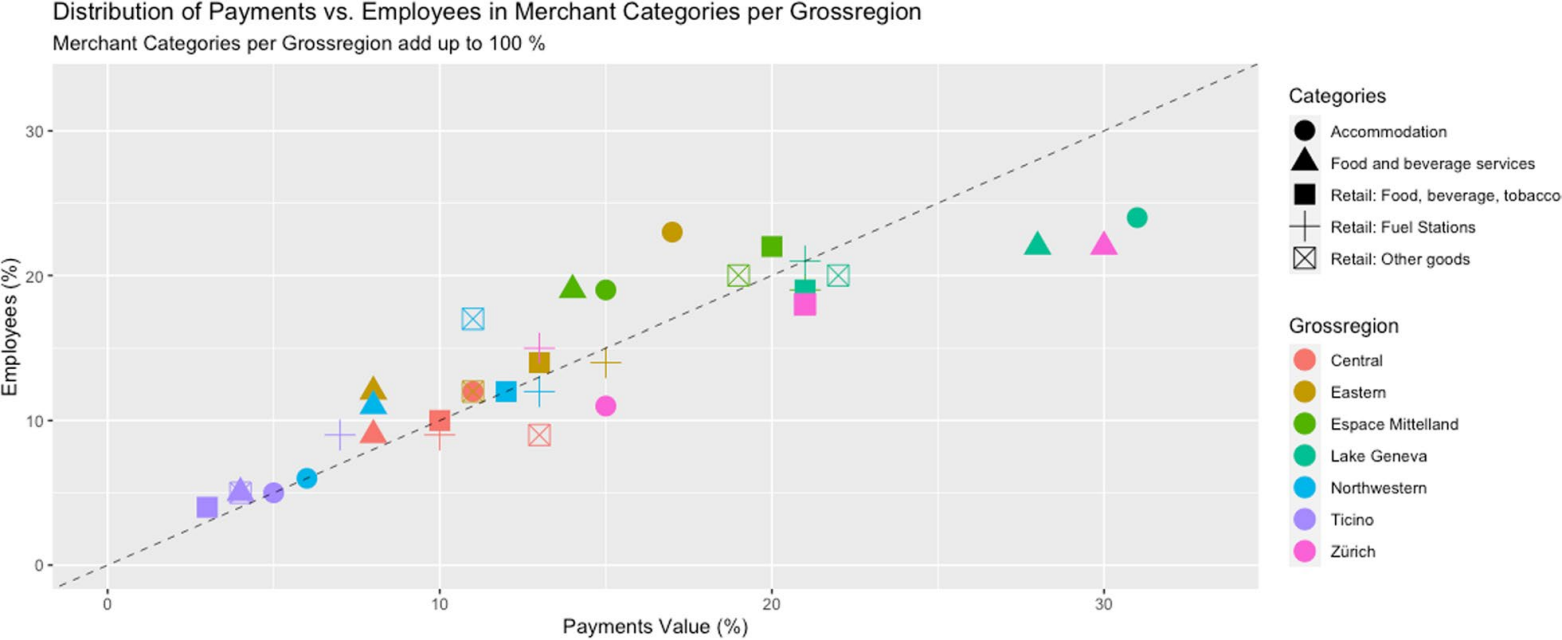
Distribution of payment turnover vs. employees by merchant category and NUTS-2 region (*Grossregion*). *Note*: The figure shows a comparison between the geographical distribution of card payment transactions by volume (acquiring data set; the figure plots total spending in 2019) and employees in Switzerland (data as per 2018). The data are displayed following the merchant category * geographic region categorization as given by the Swiss NUTS-2 regions (*Grossregion*), on a percentage basis. On the y-axis, the percentage share of employees working in a specific merchant category of the specific *Grossregion* are displayed (merchant categories per *Grossregion* add up to 100%). On the x-axis, the corresponding payment shares are displayed. The colors indicate the respective Swiss *Grossregion* while shapes indicate merchant categories. The dotted line shows the 45 degree line. Data source: ACQ_POS_Grossregion_NOGA.csv, FSO «*Arbeitsstätte und Beschäftigte nach Kanton und Wirtschaftsart*» (https://www.bfs.admin.ch/bfs/de/home/statistiken/industrie-dienstleistungen/unternehmen-beschaeftigte/wirtschaftsstruktur-unternehmen.assetdetail.23284723.html)

To further validate the representativeness of the project data, a possibility is to validate the geographic distribution of the acquiring data, exploiting the distribution of transactions across electronic payment terminals used in the project. We compare the incidence of transactions in the MCS data across regions and expenditure categories with corresponding data on the incidence of economic activity in Switzerland. The Swiss Federal Statistical Office (FSO) publishes monthly figures (STATENT) on the number of employees per merchant category and canton in Switzerland (Federal Statistical Office, 2021). When aggregated in line with the categorization of the MCS project, these data can be compared to the merchant category * geographic region categorization in the acquiring data of the project. Figure 7 shows that there is a strong correlation between the percentage shares of employees in a merchant category and the percentage shares of total cashless payment transaction volume, transacted within a merchant category for every Swiss NUTS-2 region, also called *Grossregion*.

Interpreting the correlation requires further assumptions (e.g., that the distribution of employees is approximately proportionate to the volumes of cashless payment transactions) and the comparison displayed in Fig. 7 is not exhaustive. The strong correlation in Fig. 7, however, suggests a balanced geographical and sectoral distribution of the transactions in the acquiring data sets of the project.

### Structural changes in payment behavior (cash versus cashless)

The MCS project provides data on cashless payments and ATM transactions but not on actual cash payments in stores. Special consideration must therefore be taken when analyzing the development of cashless payments in the data, as structural changes in the payment method are not adjusted for. If there is, for example, a shift from cash to cashless payments, this is not indicated in the project data but is merely reported as a positive growth in cashless payment transactions. The exact cause of this growth cannot be fully identified from the data set itself. While growth in cashless payments can be triggered by consumption growth, it can just as easily be caused by a structural shift from cash to cashless payments. It is therefore advisable to support the interpretation of the development of cashless payments with additional analysis and external data.

One possibility to obtain an indication of the shift toward cashless payments using the project data is to compare the total volume of ATM transactions (withdrawals and/or deposits) with the total volume of PoS debit card transactions (see the paragraph *Issuing Data: Cash Withdrawals vs. Cashless Payments* in Sect. 6 as an example).^20^ Such an analysis has to be done with caution, however, as further factors have to be taken into account. For example, not all cash withdrawn is actually spent, let alone spent in the region in which it was withdrawn. This leaves room for potential misinterpretation and should therefore be taken into account when analyzing the data. Moreover, shifts in the share of cash vs. cashless payments are unlikely to be homogenous across merchant categories. A reliable external source of information on shifts in payment methods—by merchant category—is the SNB's Survey on Payment Methods, which was conducted in 2017 as well as 2020 (Swiss National Bank, 2021b).

### Structural changes in data sample and representativeness

In addition to structural changes in consumer payment behavior, there are potential structural changes related to data coverage. As previously noted, the project receives data from a sample of payment transactions that are based on the debit cards issued by SIX (issuing data) or that are in the merchant pool of Worldline (acquiring data). The project data are aggregated forms of these samples where the sample size is not constant over time. Potential changes in the data provider's customer base (merchant churn) may lead to observed changes in the acquiring data. A change in the cardholder base of the issuing data will also lead to a change of the sample from which the project data are sourced and thus of the reported aggregated data statistics. An extreme example for such changes would be if the data provider of this project took over a competitor's business.

A further issue (specific to the acquiring data) are changes in consumer behavior. These might occur if consumers shift from stores with terminals of the project's data provider to stores with terminals of other payment service providers. When there are, for example, large shifts in market share within a merchant category toward stores not using the data providers terminals, this might indicate decreasing consumption in the data but is just a shift of consumption toward stores not covered by the data provider.

Finally, shifts of merchants to other merchant categories (a change in the NOGA code) might cause sudden shifts in consumption from one to the other merchant category due to recategorization. This can be a source of misleading patterns in the data.

For the researcher working with these data, this means that special care must be taken. Because changes related to the issues mentioned above are potentially worse the longer the time horizon considered, the project data are particularly suitable for analyzing short- to medium-term changes in consumption. Specifically, these issues seem less problematic when the goal is to monitor the implication of short term changes such as lockdowns or other measures in relation to COVID-19.

### Structural breaks in the coverage of transactions

One prominent example of a structural break in the data is related to ATM transactions in the data sets reporting debit card issuing data. Due to a software change, the scope of ATM transactions reported in these data sets increased from 2019.01 to 2020.09.^21^ ATMs that had not yet been migrated to the new software reported only transactions with cards not issued by the bank that owned the ATM, i.e., *other-bank* cards. Conversely, ATMs that had already been migrated to the new software reported transactions of *own-bank* cards as well as *other-bank* cards. As the software migration occurred continuously, the change in the value of transactions reported in the data sets is biased upwards.^22^

**Fig. 8:**
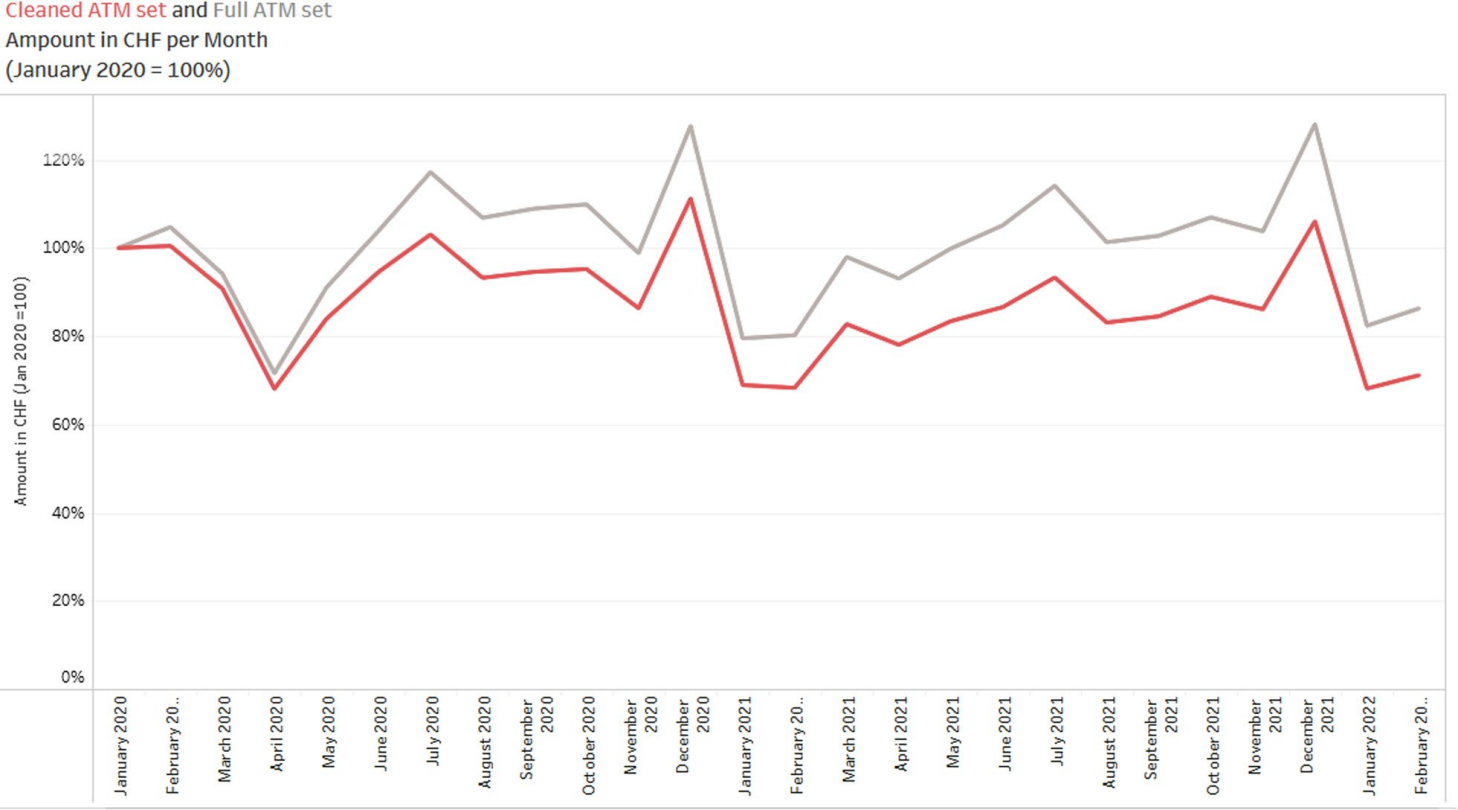
Reported ATM withdrawals in Switzerland: full vs. cleaned ATM Data Set (Jan 2020 = 100). *Note*: The figure shows the upward bias due to the change in reporting triggered by the software modification at certain ATMs. The gray line shows the raw ATM dataset without correction for the misleading reporting change, while the red line shows the data set that takes the software modification into account. Data source: ATM_Canton.csv, Debit_Canton.csv

The change in cash demand was the subject of great public and policy interest during the COVID-19 pandemic. In order to gauge the effective change in the volume of cash withdrawals, MCS publishes additional cleaned data sets for ATM transactions only. These data sets are based on a constant population of ATMs, which were already migrated to the new software before January 2020. Figure 8 compares the change in ATM withdrawals during 2020 and 2021 based on the cleaned data set (ATM_Canton) and the full data set (Debit_Canton). Our data suggest that not accounting for the change in ATM transactions, reporting would have led to a 14 percentage point upward bias in the value of cash transactions reported in September 2020 as compared to January 2020. The bias has remained stable thereafter.

## Use cases

MCS provides public access to data sets based on acquiring data (merchant view) and issuing data (cardholder view). As discussed in Sect. 3, these data sets provide complementary coverage of consumer spending within Switzerland or spending by Swiss consumers (see Tables 2, 3 in the appendix). In the following, we provide examples to illustrate relevant attributes of acquiring and issuing data respectively.

### Acquiring data: domestic versus foreign tourism

One distinguishing feature of the acquiring data is that it covers electronic payments at merchant terminals, independent of the origin of the payment instrument. For example, the data set ACQ_NOGA_CardholderOrigin.csv enables a comparison of spending by domestic vs. foreign consumers within specific merchant categories. One merchant category, which is of particular interest in this respect, is accommodation. During the COVID-19 pandemic, mobility restrictions led to a collapse of cross-border tourism. One major question for the domestic tourism sector since summer 2020 is to what extent the increase in domestic tourism (staycation) compensates for the decline in foreign tourist inflows.

**Fig. 9:**
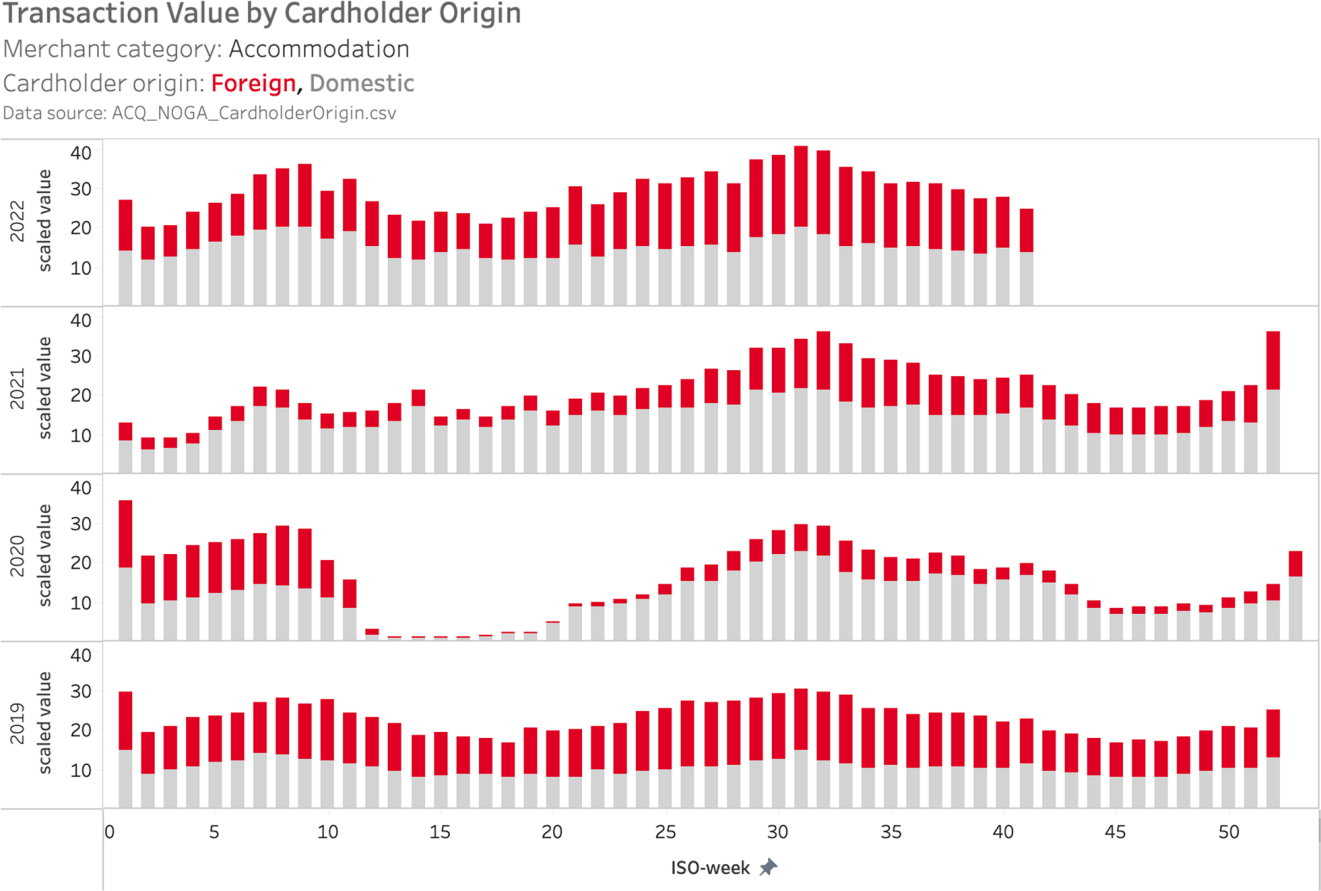
Weekly spending by domestic vs. foreign consumers on accommodation in Switzerland, 2019–2022. *Note*: The figure displays scaled total weekly spending with debit cards, credit cards and mobile payments at merchants in the hotel category for 2019–2022 from the acquiring data set. Spending by domestic cardholders/users is reported in gray, spending by foreign cardholders /users is displayed in red. We display the data as provided, i.e., as a scaled value, which preserves the relative interpretability both over time and in the cross-section. See Sect. 2 for further details. Data source: ACQ_NOGA_CardholderOrigin.csv

Figure 9 shows that during summer 2019 foreign consumers accounted for 55%-60% of electronic payments in Swiss hotels. This share dropped to 20–25% in summer 2020 and then recovered to 35–40% in summer 2021. Overall, spending between mid-July and mid-August only declined by 5% from 2019 to 2020, and then rebounded by 21% from 2020 to 2021. As discussed above, however, the comparison across years is hampered by the shift in payment methods from cash (not observed in this analysis) to electronic payments.

### Acquiring data: PoS versus ecommerce in non-food retail

A second notable feature of the acquiring data is that it provides information on the channel of consumer spending, i.e., ecommerce vs. PoS. This data feature is especially interesting in light of the lockdown of (non-essential) physical sales outlets and the shift to online shopping during the COVID-19 pandemic.

**Fig. 10:**
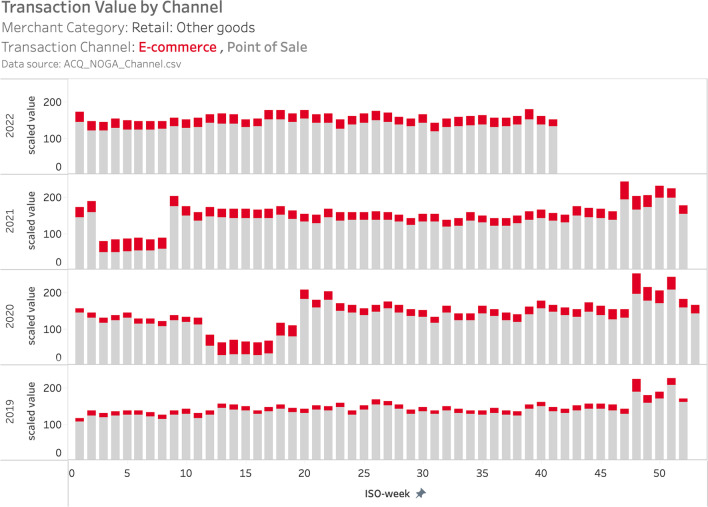
Ecommerce vs. PoS spending in non-food retail. *Note*: The figure displays scaled total weekly spending with debit cards, credit cards and mobile payments at merchants in the Retail: other goods (= non-food) category for 2019–2022. Spending at PoS terminals is displayed in gray, spending at ecommerce terminals is displayed in red. We display the project data as provided, i.e., as a scaled value, which preserves the relative interpretability both over time and in the cross-section. See Sect. 2 for more details. Data source: ACQ_NOGA_Channel.csv

Figure 10 illustrates consumer spending in non-food retail by week over the period 2019–2022 from the acquiring data set. For the period of the first lockdown (ISO weeks 12–17, 2020) the graph shows a 55% year-on-year decline in total spending. However, during this period ecommerce spending almost tripled, so that its share in total non-food spending increased from 8% in 2019 to 53% in 2020. A similar, albeit less pronounced shift from on-premise to online shopping occurred during the second national lockdown in ISO weeks 3–8 in 2021.

### Issuing data: cash withdrawals versus cashless payments

The issuing data sets published by MCS cover transactions by debit cards issued by Swiss banks only. Debit cards account for 33% of non-recurring spending by Swiss consumers (SNB 2021). However, importantly, a further 24% of non-recurring spending is made in cash, with the overwhelming majority of cash withdrawals conducted at ATMs using debit cards. This implies that the debit card issuing data allow insights into the change in payment behavior from cash to cashless payments over time. Recalling our discussion in Sect. 5, we emphasize that cash withdrawal data must be interpreted with care because there are many factors that affect cash payment behavior but cannot be controlled for with our data.

Caution is also advisable with such an analysis because further factors have to be taken into account, such as the fact that not all cash withdrawn is actually spent, let alone spent in the region in which it was withdrawn. This leaves room for potential misinterpretation.

**Fig. 11:**
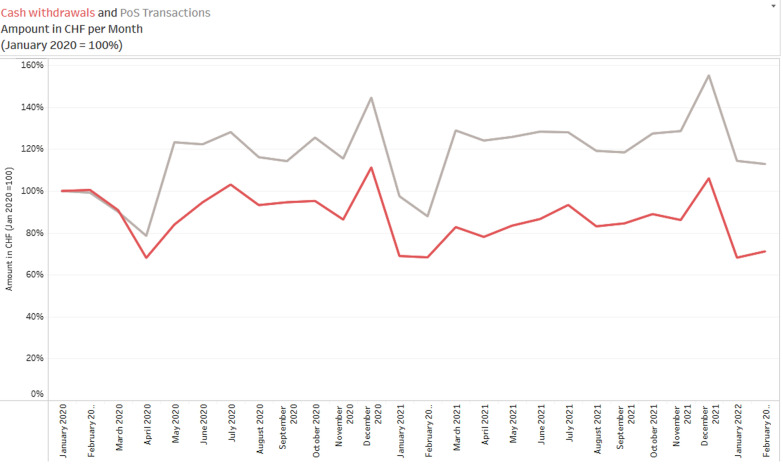
Debit cards: ATM withdrawals vs. PoS transactions. *Note*: The figure displays monthly spending (in CHF) with debit cards issued by Swiss banks for 2020.01–2022.02 from the project data. Spending at PoS terminals is displayed in gray, cash withdrawals from ATMs are displayed in red. Cash withdrawal transactions are based on a constant population of ATMs, which report all transactions of own-bank and other-bank clients from 2020.01. See Sect. 5 for details. Data source: ATM_Canton.csv, Debit_Canton.csv

Figure 11 documents a 14% increase in PoS transactions by debit card in January 2022 compared to January 2020. By comparison, ATM cash withdrawals by the same population of debit cards declined by 32% over the same period. Cashless payments have increased significantly since the onset of the pandemic at the expense of cash withdrawals.

### Issuing data: cross-border spending

A second unique feature of the debit card issuing data is that they allow to track the spending behavior of Swiss consumers abroad. Thus, the issuing data can provide insights into cross-border vacationing as well as shopping tourism by Swiss consumers. The data set Debit_Merchanttype_Grossregion.csv, for example, provides breakdowns by merchant category and country, allowing to separate spending likely associated with vacations (accommodation, food and beverage services) and spending likely related to cross-border shopping (food retail, non-food retail). The same dataset also covers ATM withdrawals by Swiss debit card holders abroad.

**Fig. 12:**
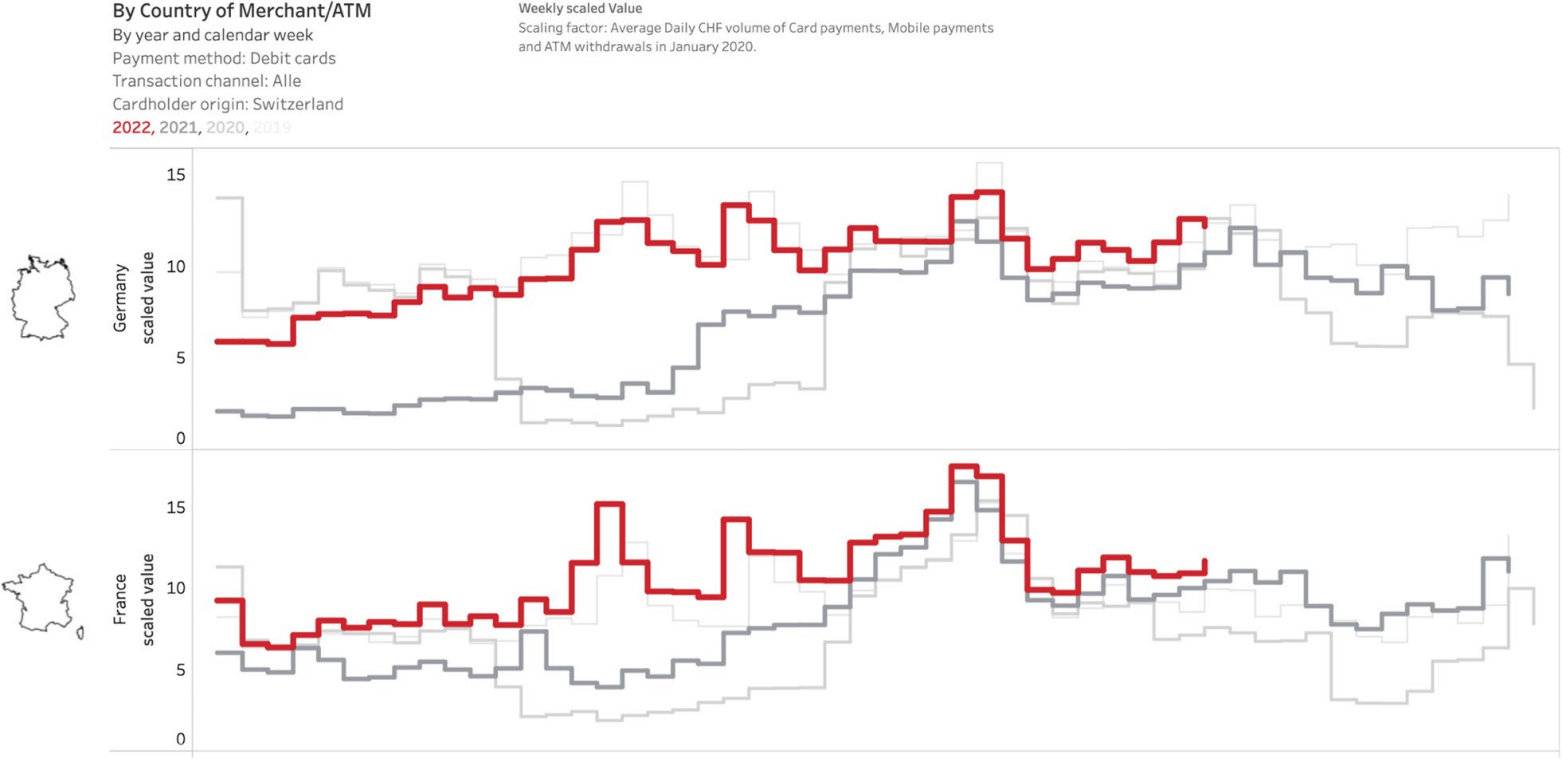
Cross-border debit card transactions by Swiss consumers in Germany/France. *Note*: The figure displays weekly scaled payment transactions with debit cards issued by Swiss banks for 2020–2022. Transactions include payments at PoS terminals and cash withdrawals at ATMs. Spending in Germany is displayed in the upper panel, spending in France in the lower panel. We display the data as provided, i.e., as a scaled value, which preserves the relative interpretability both over time and in the cross-section. See Sect. 2 for more details. Data source: Debit_Merchanttype_Grossregion.csv

Figure 12 documents total cross-border debit card transactions by Swiss consumers in Germany and France by week from 2019 to 2022. We observe a sharper decline in spending in Germany compared to France during the lockdown periods of spring 2020 and early 2021 when cross-border shopping was not feasible (because borders were effectively closed). We also observe a stronger recovery of spending in France during summer 2021, most probably related to increased numbers of Swiss vacationing in France.

## Impact and outlook

From its beginning, the aim of MCS was to not only provide the public with timely and meaningful data on Swiss consumption but to offer first-hand insights and analyses on evolving economic policy debates. To date, the data published by MCS have been referred to or covered in more than 300 news reports and 12 radio and television contributions.^23^ Examples of well-covered topics in the media are the decline of foreign tourism and increase in domestic tourism (staycation) in Switzerland since 2020, the impact of mobility restrictions on cross-border shopping of Swiss consumers, or the impact of vaccination certificates on restaurant turnovers. Extended analyses of the data have been published in policy blogs and policy journals (Brown et al., 2020a, 2020b). Moreover, several researchers have made use of MCS data in policy papers related to the COVID-19 pandemic (Alvarez & Lein, 2020; Eckert & Mikosch, 2020; Kraenzlin et al., 2020; Seiler, 2020).

Real-time data on consumer spending have greatly improved our knowledge on the state of the economy. MCS will continue to track the spending patterns of consumers in Switzerland, documenting the effects of shocks and (economic) policies on consumption expenditure. Real-time spending data offer numerous new avenues for research or prediction. The MCS team supports related initiatives by providing data and advice to those who intend to create new insights through data provided by the project.

## Data Availability

The datasets generated and/or analyzed during the current study are available in the Switchdrive repository, https://drive.switch.ch/index.php/s/yLISs3KVE7ASE68

## Appendix

See Tables

**Table 2:** Data sets based on acquiring data

Acquiring data set (.csv)	Payment terminal attributes			Payment instrument attributes	
	Location	Merchant category	Channel	Type	Origin
ACQ_CountryOrigin					Country of origin
ACQ_NOGA_CardholderOrigin		12 categories			Domestic vs. Foreign
ACQ_NOGA_Channel		12 categories	Ecommerce vs. PoS		
ACQ_NOGA_PaymentMethod		8 categories		Debit /credit/mobile	
ACQ_POS_Agglomeration_NOGA	7 Agglomeration types	7 categories	PoS only		
ACQ_POS_Canton	26 Cantons in 7 regions		PoS only		
ACQ_POS_Grossregion_NOGA	7 NUTS-2 regions	7 categories	PoS only		
ACQ_Transaction_Type			Ecommerce vs. PoS	Debit /credit /mobile	Domestic vs. Foreign

2,

**Table 3:** Data sets based on debit card issuing data

Issuing data set (.csv)	Payment terminal / ATM attributes		
	Location	Merchant category	Channel
ATM_Agglomeration	7 Agglomeration types		ATM only*
ATM_Canton	26 Cantons in 7 regions		ATM only*
Debit_Canton	26 Cantons in 7 regions of Switzerland & Transactions abroad by country		PoS vs. ATM
Debit_Merchanttype_Agglomeration	7 Agglomeration types	12 categories	PoS vs. ATM
Debit_Merchanttype_Grossregion	7 NUTS-2 regions of Switzerland & Transactions abroad by country	12 categories	PoS vs. ATM

Data marked with * are available from 2020.1.1 only. These data sets are based on a constant sample of ATM’s in Switzerland, which had been migrated to the “ATMfutura” software as per 2020.1.1. Data set includes withdrawals from and deposits at ATM. See Sect. 5 for more details

3 and

**Table 4:** Overview of attributes in the acquiring and issuing data

Attribute	Acquiring data	Issuing data
Date of transaction (daily frequency)	X	X
Agglomeration type	X	X
Region: canton or Grossregion	X	X
Channel: PoS/Ecom	X	
Payment method: credit/debit/mobile	X	
Transaction type: ATM/PoS		X
Merchant category: NOGA	X	X
Cardholder origin: domestic/foreign or country level	X	
Country of transaction		X
Scaled value	X	X
Scaled volume	X	X

“x” indicates the availability in the respective data set

4.

## Footnotes

1 Strictly speaking, the data are therefore not real time. The fact that similar macroeconomic figures are typically available at a monthly or even quarterly frequency motivates our wording.

2 www.bundesbank.de/wai.

3 https://www.oenb.at/Publikationen/corona/bip-indikator-der-oenb.html.

4 Index zur wöchentlichen Wirtschaftsaktivität. See also Wegmüller et al. (2021).

5 As a mobile payment instrument we categorize payments via mobile payment apps that are mostly directly debited to a bank account (e.g., Twint, Alipay), but not transactions that are made with a mobile device but mostly charged to a credit card (e.g., Google pay or Apple pay).

6 Because confidentiality prevents us from mentioning the coverage in percent, in Sect. 5 we provide further comparison of the type of transactions covered by the project’s acquiring and issuing data with the SNB payment statistics.

7 At the time of proofreading, the data contributed by SIX are provided until the end of 2022 whereas data provision by Worldline is ongoing.

8 The corresponding mapping table is available at the project website in the data folder “4_Mapping Files”.

9 https://en.wikipedia.org/wiki/NUTS_statistical_regions_of_Switzerland.

10 https://www.bfs.admin.ch/bfs/de/home/grundlagen/raumgliederungen.assetdetail.349566.html.

11 In some of the dashboards on the website we use three coarser agglomeration categories. The category *City Centres* contains core cities; the category *Agglomeration: Other* contains core municipality of agglomeration: primary core, core municipality of agglomeration: secondary core, municipalities oriented to multiple cores; and the category *Other Municipalities* contains rural municipalities without urban character, core municipalities outside agglomeration, and the residual category *Other* mentioned in the text.

12 See Sect. 2 for an explanation of the data scaling.

13 We can only distinguish between PoS and ecommerce transactions in credit card data, not in debit card data.

14 https://public.tableau.com/en-us/s/.

15 https://cran.r-project.org/web/packages/anomalize/index.html.

16 https://cran.r-project.org/web/packages/forecast/index.html.

17 We choose a relatively short period because we are mainly interested in estimating the weekly seasonality at a good level of precision, while adapting to some degree to potential structural breaks in the data, e.g., induced by behavioral shifts or policy measures.

18 The MCS project offers a visualization of the SNB data on the dashboard *Monthly Payment Card Transactions*. The original data are available on the SNB data portal: https://data.snb.ch/de/topics/finma#!/cube/zavezaluba.

19 As mentioned previously, only credit card data are flagged for PoS and ecommerce transactions.

20 Another example is the featured analysis of MCS project available at the “Featured Analysis” tab on the project website.

21 As documented in the aggregate monthly payment data of the Swiss National Bank (https://data.snb.ch/de/topics/finma#!/cube/zavezaluba) this change in reporting started in May 2017, with the largest increase in covered transactions occurring in 2019.

22 https://www.six-group.com/en/newsroom/media-releases/2020/20201006-atmfutura-implemented.html.

23 An updated list of all media reports based on MCS data is available at the “Media” tab on the project website.
